# Supplementary material for: Distinction of Paramecium strains by a combination method of RAPD analysis and multiplex PCR
Source: PLoS One. 2022 Mar 11;17(3):e0265139. doi: 10.1371/journal.pone.0265139 (PMC8916638; doi:10.1371/journal.pone.0265139)
Supplement: S1 File — (PDF) [file pone.0265139.s001.pdf]

DNA sequencing result 1

Template DNA: No.13, Primer: 02, Pc_1 primers were designed from this sequence (underlined)

GAGGGATCCGAATTGCAGGATACGGGATATATGTACATGTGAATGGTGCCAAATAC
GAAGGGTATTGGAGGAACGACCTTCAAGATGGTTATGGGATTGAGACTTGGGCAG
ATGGTAGTAAGTATGAAGGGTTTTATGCAAATGGGAAGAAAGATGGTTAGGGAAAA
TATGAATGGCCTGATGGTAGTAAGTACAATGGTCTATGGAAAGAGAATTAAATAGAT
GGAATGGGTACATATGAATGGTAGGATGGAAGGAAGGTATTTGAGCTTATTAATTAG
TATTGTGGAGAATGGCAATAAAATTTTATGCATGGAAAAGGTAAATATATTTGGAGG
GATGGTCGTCAGTATGATGGAGAGTTTTAGTTAGATAAAAAATCAGGCTTTGGGGT
ATATGTGTGGGAGGATGGTAGAAGATATGAAGGAATGTGGGAAAATAACAAACAGC
ATGGAGAGGGTAAATACTACGAACCAGACGGAGTGATGAGAAGAGGCTTATGGGA
AGATGGAAAAAGAGTAAAATGGGTTGACGAGTGACATATATAACACTATCTCAAATC
AAAGAATCGAGTGAGGCTGAGGCTTCTTGCAGCAAATTTAAAATAAGGGCTTTGCA
TTCNTCTACTCCATCTTCGCTGAATTGAGCCAAGTGTTCTTTTATGTAGAGAACAAA
TTCATTTTCCTGTGATTCAGTAAGTTTTTCAGCGACATAGACAAGCCATTATTCGTC
AGATGTGTAGTTTTTATCTCTAAAATTGATGAGATGTTTAATCTTTTAGGTGTACTCA
ACTTTGGCTTTCTTTTGTTGATCCATGAGGATGGCATCGTACTTTGATTGTAGTTAT
TCATCTCGAATAGAGAAGTATGTATAACTTTGCAAAAATTCTGCCCTCAATTCTTGG
AGTTCTTATTTTATGTAGTTTTGAGTAAAGAATTAATCAAAGTTATCTAAAGTAAGTA
CTCTTAGGGCATCATCAACTTTTCTTTGTAATGTTTCCAATTCATAAACTCCTATGCT
TCTTAAACATTTTGAGCAAGCTGATACAATTTTTGAAGAATCTTTCTCCTTAACTGG
CATTCTGTAAATGGCTGGGCAGTTGTGAAGGGAGCATTTAGGTTGATAAACAGATT
TCCTATCTGGTATGATATCTGACATATCATTTGTCGAAAACATAAGAGGTGACATTG
CTTATTATTAATAGAGTGTTTGTGGTTAAAAGGAAAGTGAATTGGATGAGACATTCT
ATTTGTATTGTCTTATCATATCAATGACCCATATCAGGGCCTTTCGATTGGTAGGTTC
TTTCTGTATCATACCAGTCAGAATGCTTGCTATGATTGATTTACGATTTGGTAATCTA
CTTGGCTTATATTCAGCCTTGCGAGGATAATATGGTTCATTTCTAATTTTAATGAAGA
CTTCATCTTCTGACATACCATCATAAAATGGTGAGCCATGTAATAATTCATCAAACAT
TAACCCTAAGGCCCAAACATCAACTGTTTTATCGTATCCTGCAATTCAAGCTGTCGA

DNA sequencing result 2

Template DNA: No.12, Primer: 05, Pc_2 primers were designed from this sequence (underlined)

GAATTCTAGCTCTGGACAGAATAGTGACTCTTCTTGCATGAACTGCACATAAATTGG
TGTCTTCAAATAATCCAACTAGATATGCTTCAGTAGCTTCTTAAAGAGCTATAACAG
CTGAGCTTTGAAATCTGAGTTCCTTTTAGACTTAGCTTGCTATTTCTCTAACCAATC
TTTAGAATGGAAGCTTTCTGATTAAGAGTTGAGTGGACTTTTAGTATTTTCTAATCT
CCCTCAAAGCAACAGTTCCTGGTCGAAATTTGTGTGGTTTCTTTAAACCTCCAGAT
ACTGGGGGTGTGGAGTAATATTTTATAGGTTTCTTAGGAGTGTTGTCTCTCGGTGG
TTATTTGATTCTTGCCATTATTATTAAGTTGATTTTTTGATCAATTGTTATTGATTAAT
TGATGCATTAGTACCTCATTACCTACGTTAAAATCATGAATCTTATCTCATATAAATT
TAATCTAATTTGTCTAAAGATTTTATCAATCCTATGAATGCAAACATGAATATTTTAA
GACGTAAAATTAAATAATTAATGGAAGCCAAGAAGATCATCGGATTAGGAAGTCCC
CTGTTAGATATTCAAGCTGAGGTGTCTGCTGAGTTTTTAGAGAAATATGGCTTAACC
CTCAATAACACATATTTCGCTGAAGAAAAGCACATGCCATTGTATGAAGACTTAATT
AACATACCAACTCACTCTCACGTCCCAGGAGGTAATATACCATTTAATGAGGTTCCG
CACTTAACACCATTCGACTTGCAAGATGGATGGCCCAAGCAGGACCAGAATAGGTG
AAATTCATCGGATGCGTCGGAAAAGACAAATTCGCTAAAATGCTTATCGAAGTCAC
CAATTCAGATAGTGTCACAACATTATTCGATGAATAAGACTAACCCACAGGAAAGTG
TGGTGTGTTGTTATGTAATAAGGACAGGTATAATTTCATATAACAAAGGTGTTTGGT
TCCATTGATTGGTTCAGCTGCACATTTATCGTAAGAGTATGTCGAATAACACATCAA
TGACATCAAAACAGCCACCGTCTTATTCAGTGAAGTGTATTTCCTTTATCCCAGAGC
TAGAATTC

DNA sequencing result 3

Template DNA: No.12, Primer: 02, Pc_3 primers were designed from this sequence (underlined)

GGGCCCTCCGCGGGTCTGGTGCCACGCGGTAGTGGTGGTATCGAAGGTAGGCATA
TGGAGCTCGGTACCCTCGAGGGATCCGAATTGCAGGATACGGCAATGTAAATGCTG
AATGTAAAAGATGCACTACAAATTGTGATAAATGTTTTGGTATAGGTCCAGATGAGT
GTACAGATTGCTCAAGTGGATATTATTTCCTCCACAACTAATGTTATGGTAGATGTC
CTAAAAATTATGTTGGAATAAGACCATAATATATGTGTAAGTGCATATTTGAAAATTG
TGTATCTTGTACAGAGACATAATTCAATCTTGATAATGTATGTTATGATAAATGTCCT
AATGGAACATTTGGATTTAATGGATTATGTATAAAGTGTGATAAAAGTTGTGGAACT
TGTTCAGGAGAGAGTTTTGATTAATGTGACTCATGTAGTTTTCCTTTAATCTTTTATT
AACACACCTGCCTTAGTTAATGTGAAAACAATACATATCATGATATTACTACAAATG
AATGTCTTTTATGCGATGATTCTTGTTTAGCTTGTACAGGACCACATTTAATTGATT
GTACTGCTTGCAAAAATGAATAATTGTTAAATGTTGATGGATCATGTTAGGACTAAT
GTACATCTGATTCATATGTTATTATTTCTGAAAGAAGATGTTTAGCATGTCATCCAAC
TTGTTTAACATGTTTTGGTGGGATGATTAATAATTGTTTGACATGTAAGAAATTAATT
TATTTGAATGAATGTGTTGATACGTATCCTGCAATTCAAGCTTGTCGACCTGCAGTC
TAGATAGGTAATCTCTGCTTAAAAGCACAGA

DNA sequencing result 4

Template DNA: No.13, Primer: 04, Pc_4 primers were designed from this sequence (underlined)

GAATT<u>GCAGAGAAGGGTAATACTTC</u>ATTAGTTTTAGATAGATCACCACCTAAATAAG
TATGTATAATACCTTTTGTATCTAAGTAGGCTTTAAATTGATTTACTGCTTATTTATCA
ATTAATCCTTTTCTCATTGACAATGGATGAATAGGGTAGATGAGAAAATAGATTTAA
ATATAAAACTCCAACTTCCAAGAATCATGATCTTATCGTTATGAGATTGGGATATAG
CGACTCCAAGATTCAAGAAATTTATCTCTTTCACCCCTATCAAAATGTCCAAGAATG
ACATATTCTGTTTCACTTCCCTATTCAGTTGCAAATTATTATTCTATCCGAAATTGTT
CCAATGTTTTGAAACAATTACATCTAACCAAATAATCCTATACAATATTGTTCAATTG
TTCCACTAAATTTCCTCCTTAATCAACTGATTTATCAGCCTAAATATAACAAATTGTT
ACAGATTATACAGCAGATTGAACTCTCTTTTATGGAGGGTCTACTTTGGAATTGCGA
CTTAATCCAGAATTTACTTTA<u>GCTCTTACATCCTTCTCTGC</u>AATTC

DNA sequencing result 5

Template DNA: No.13, Primer: 09, Pc_5 primers were designed from this sequence (underlined)

GAATTCGCTGTTACCAATTAGACATCATGGAAGAAATACTGGGGAGGTGCAGGAAT
ATCGGTTGATATTTTTACGAATTCCTCCTCAGTTATGTTTTATAATGCCCAATTCTTA
GCCTTCTACTCACCGATAGTGCTAAATGTTCCAGCGCCTATAGCTTTCCCACATGCT
GATCCAGCCCCATGTCCAGGGTATAGGACTACATCATCATTGAGGGTTATGACCTT
GTCCCTTAATGAATGGTAGAGCAGGGAGGCAAGCTTTTCTGTTGACAAGCCTGTAG
CTCTTGATGCTAGGTCTGGTCGACCAACTTCTTCTAGGAACAAAGTGTCTCCACTG
AATACACATCGATCCTTGCCTTCATCGACCAATACAAAGCAGCTTGATTCGAGTGTA
TGCCCTGGAGTGTGTAGTACTCTCAGTTGCACATGTCCAAGGGGTAATAATTCTTTA
TTATTCCTAATAGTACCTTCGTCAGAAGCAATTTTGGCTTCATACTTGGTAACAGCG
AATTC
