# Supplementary material for: Distinction of Paramecium strains by a combination method of RAPD analysis and multiplex PCR
Source: PLoS One. 2022 Mar 11;17(3):e0265139. doi: 10.1371/journal.pone.0265139 (PMC8916638; doi:10.1371/journal.pone.0265139)
Supplement: S1 Table — (PDF) [file pone.0265139.s002.pdf]

Quick identification chart for each standard strain

Name of standard strains	Method	Result	Approximate size	Related figure
P. caudatum<br>dKNZ-12O	Multiplex PCR	Pc_1 (-)<br>Pc_2 (-)<br>Pc_3 (-)<br>Pc_4 (+)<br>Pc_5 (+)	-<br>-<br>-<br>554 bp<br>553 bp	Figure 4B and 5
P. tetraurelia<br>51	RAPD PCR analysis<br>(Random primer 02)	Single band	3000 bp	Figure 2A
P. bursaria<br>Yad1g1N	RAPD PCR analysis<br>(Random primer 03)	Two distinct bands	4000 bp<br>2000 bp	Figure 3B
