# Supplementary material for: Distinction of Paramecium strains by a combination method of RAPD analysis and multiplex PCR
Source: PLoS One. 2022 Mar 11;17(3):e0265139. doi: 10.1371/journal.pone.0265139 (PMC8916638; doi:10.1371/journal.pone.0265139)

Figure 1A
DNA: *P. caudatum*
Primer: 02

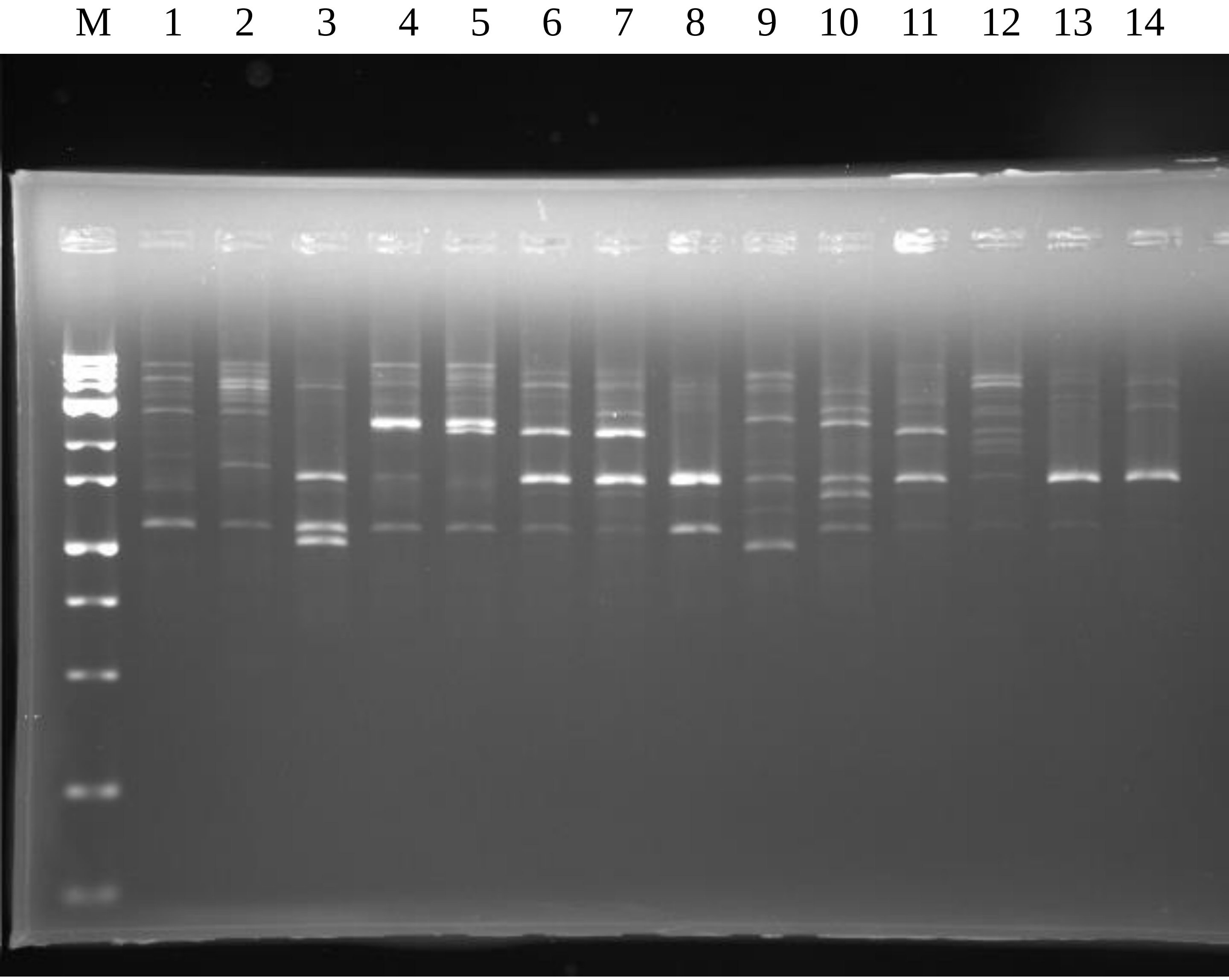

Figure 1B
DNA: *P. caudatum*
Primer: 03

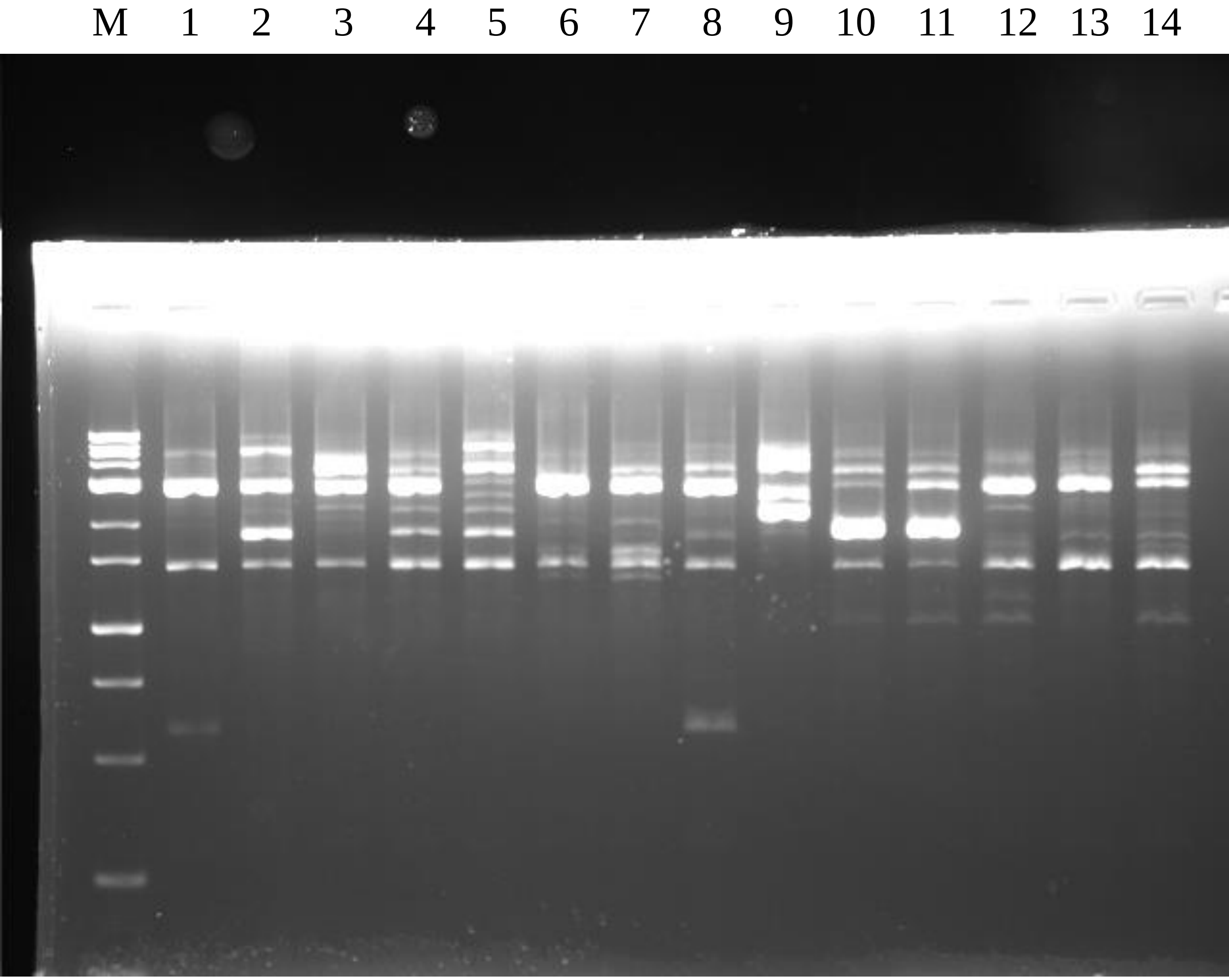

Figure 1C
DNA: *P. caudatum*
Primer: 05

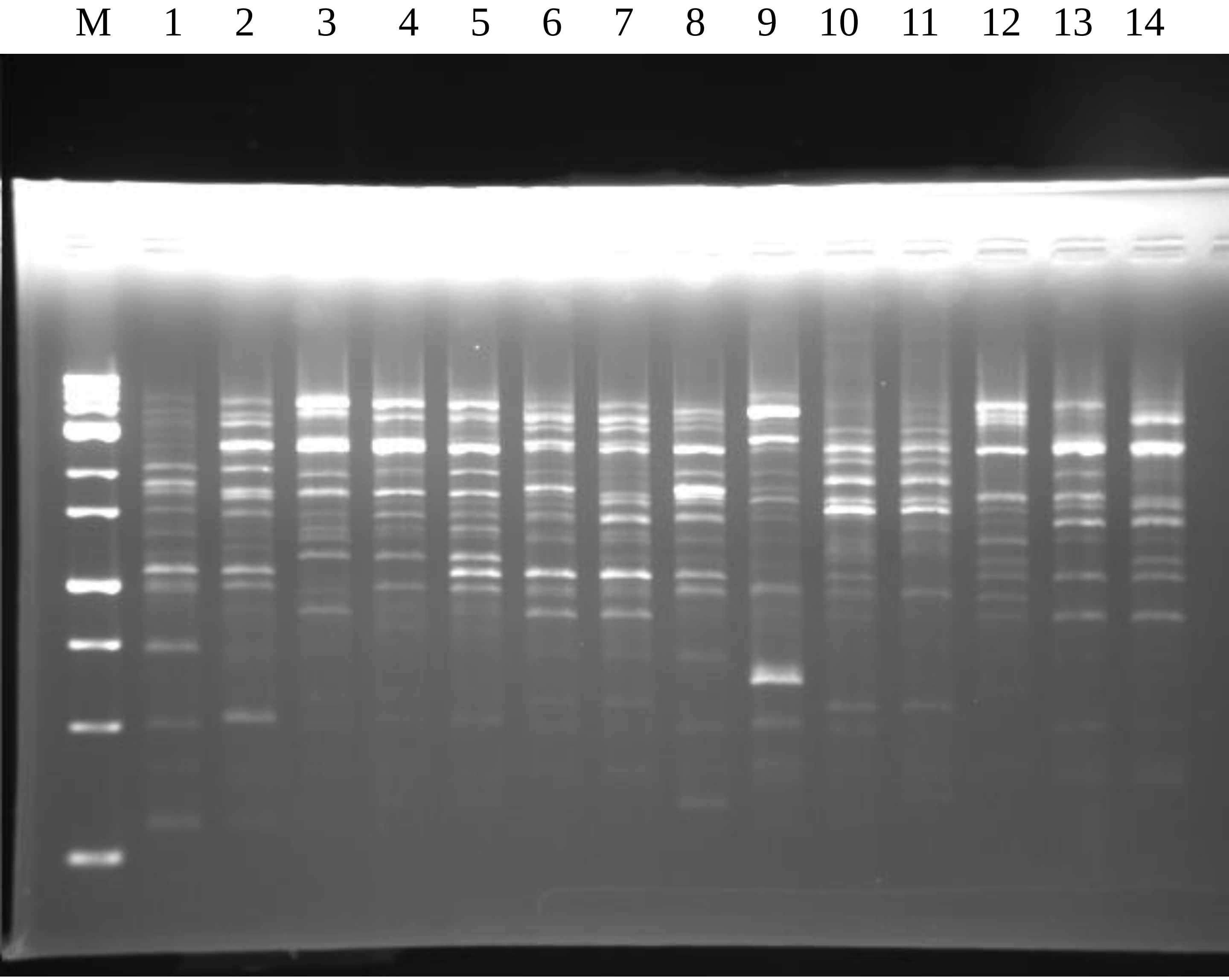

Figure 2A
DNA: *P. tetraurelia*
Primer: 02

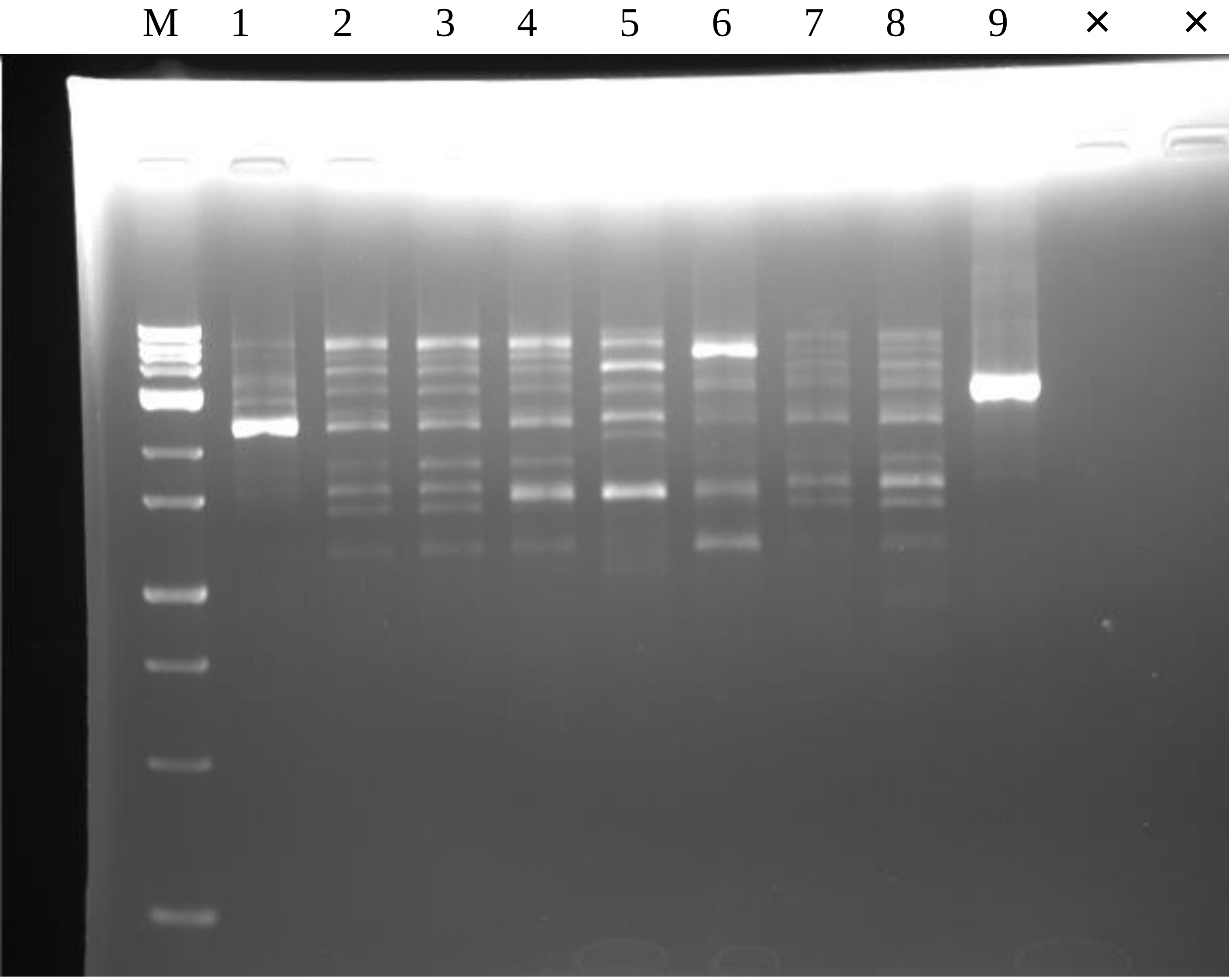

Figure 2B
DNA: *P. tetraurelia*
Primer: 03

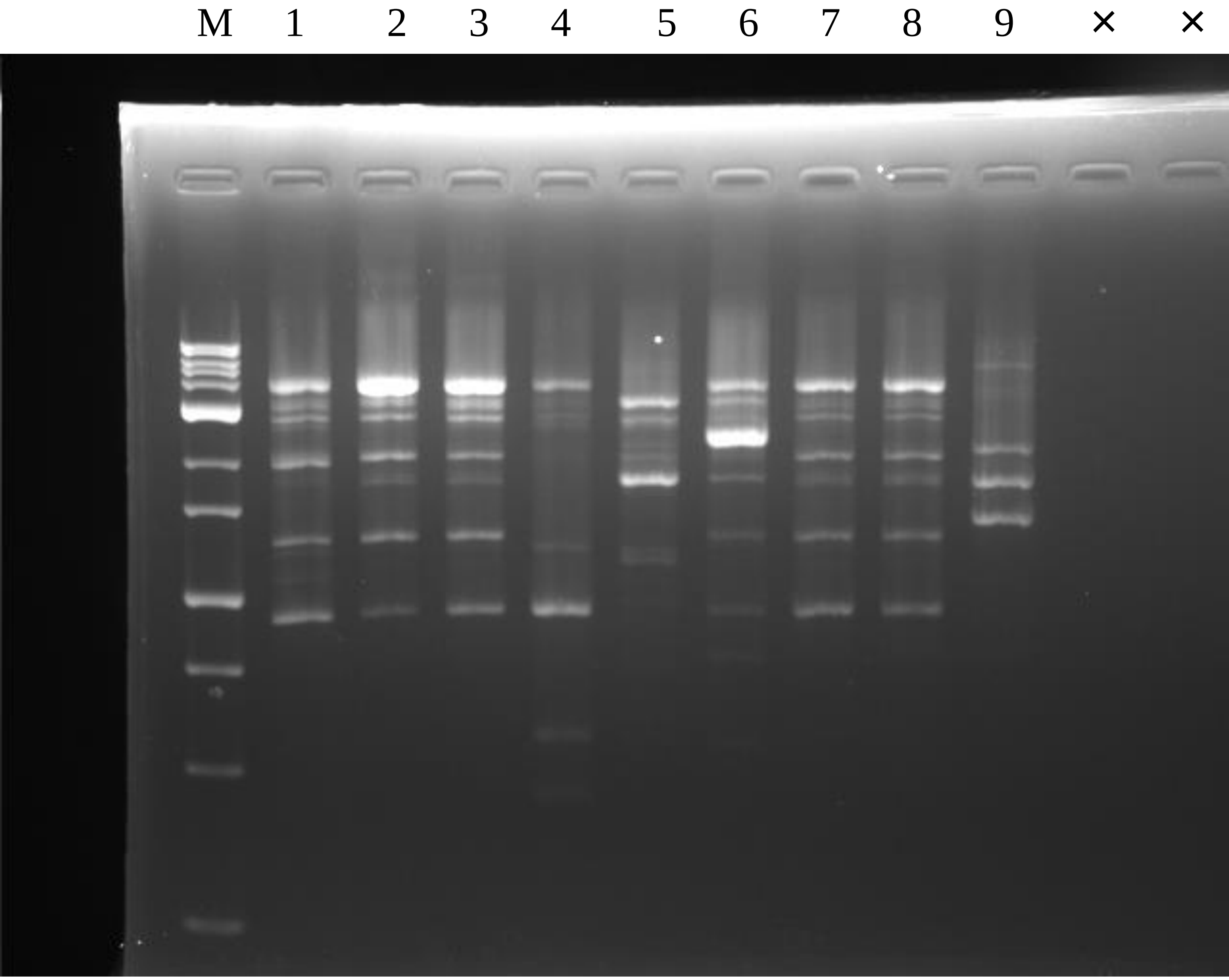

Figure 2C
DNA: *P. tetraurelia*
Primer: 05

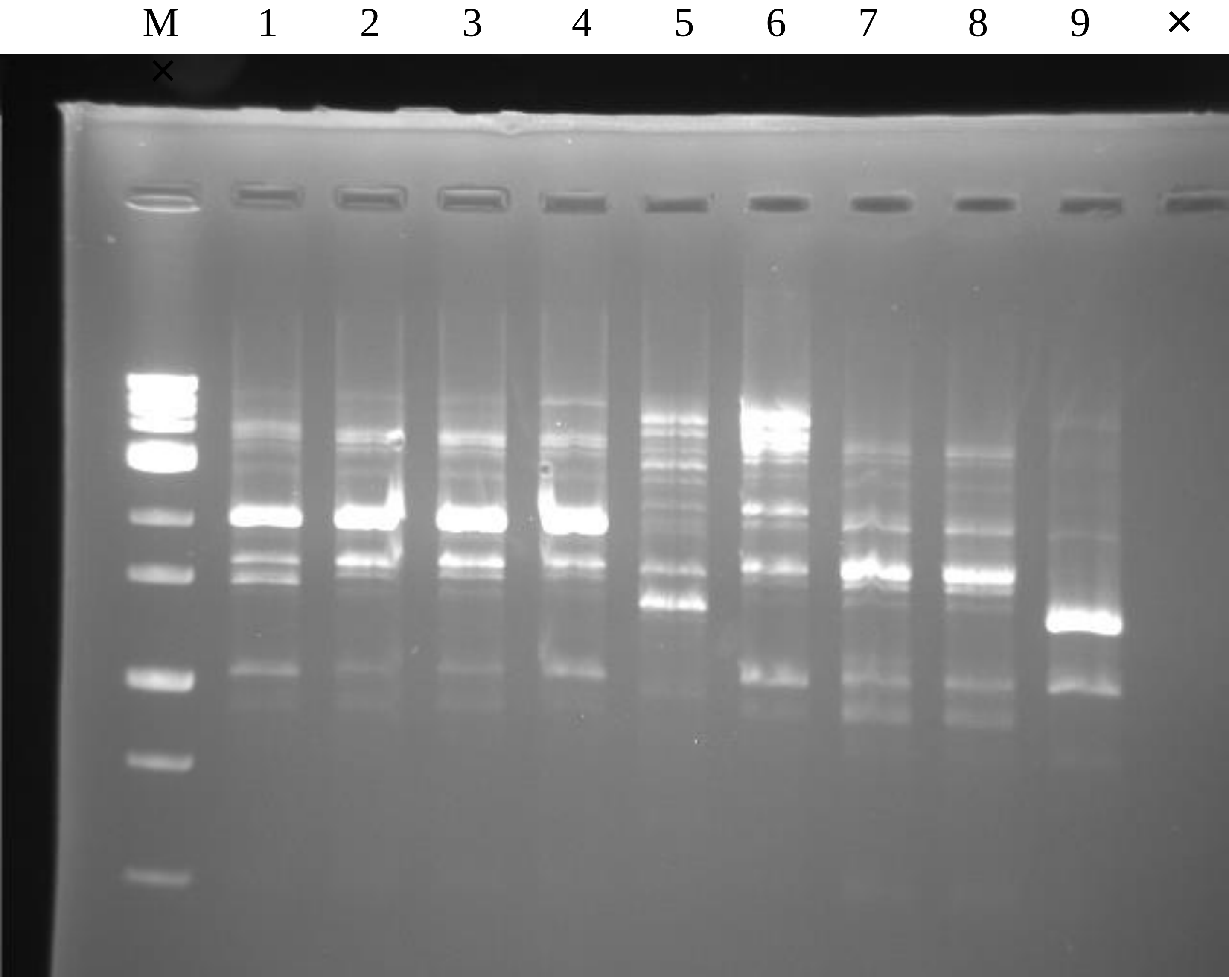

Figure 3A
DNA: *P. bursaria*
Primer: 02

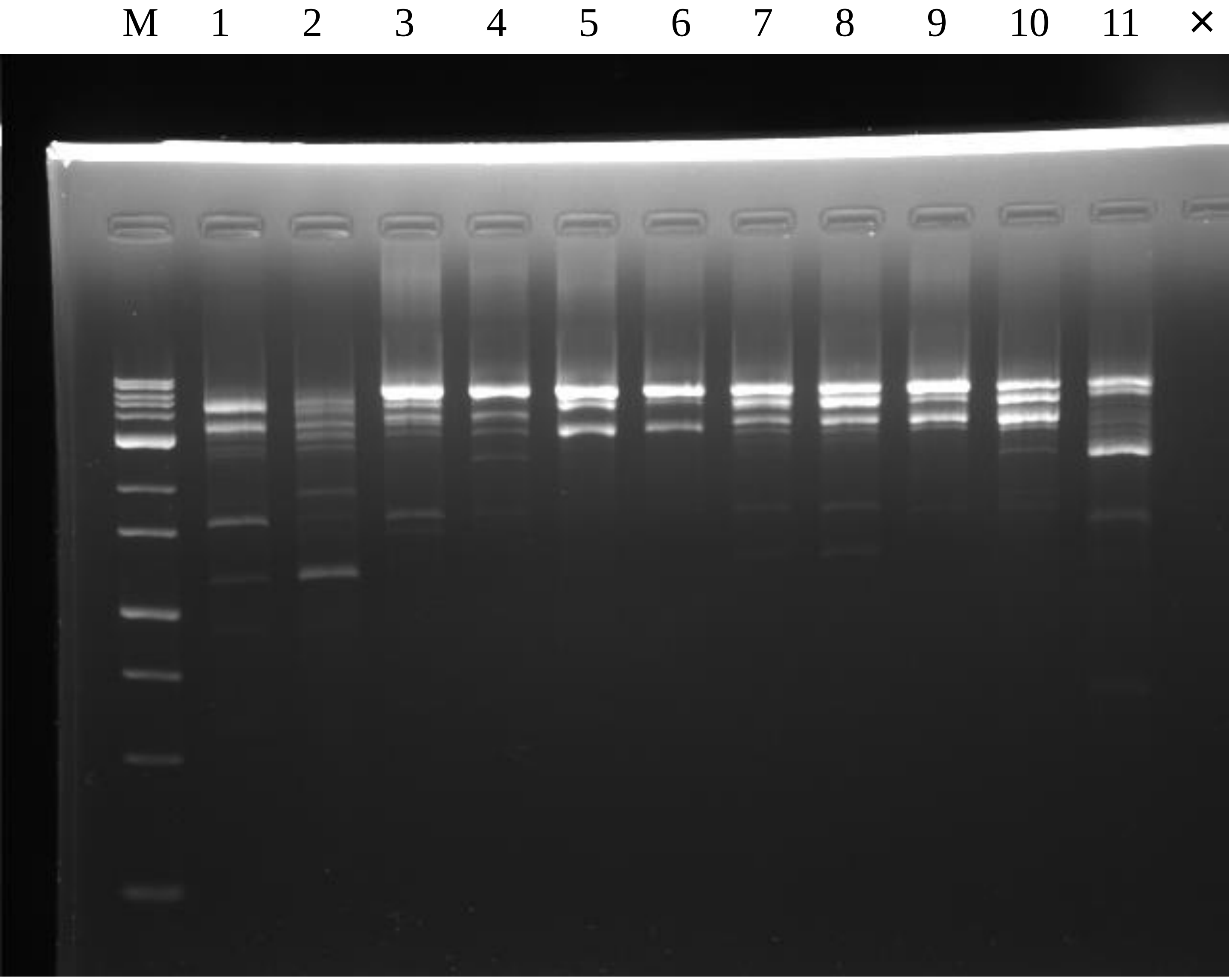

Figure 3B
DNA: *P. bursaria*
Primer: 03

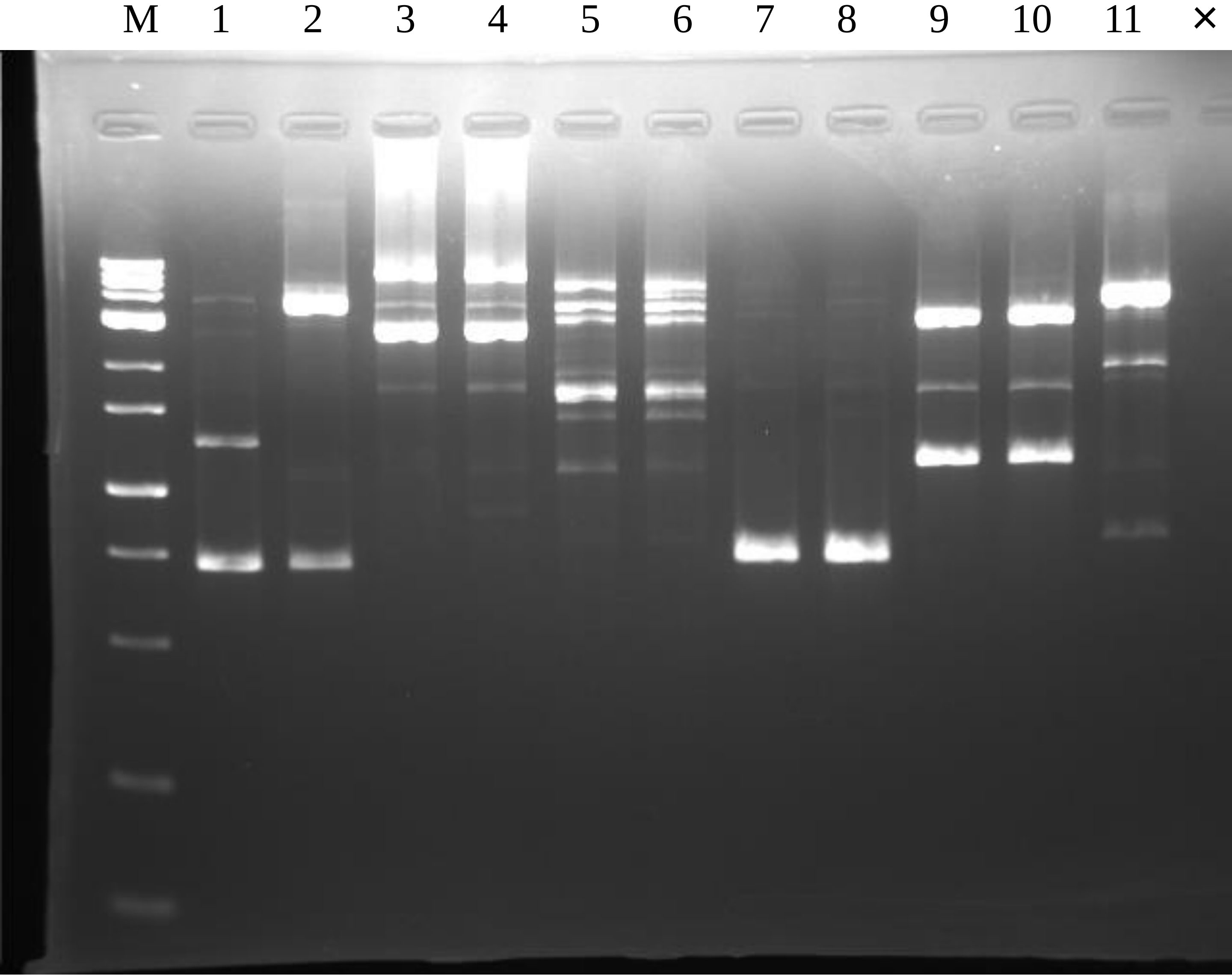

Figure 3C
DNA: *P. bursaria*
Primer: 05

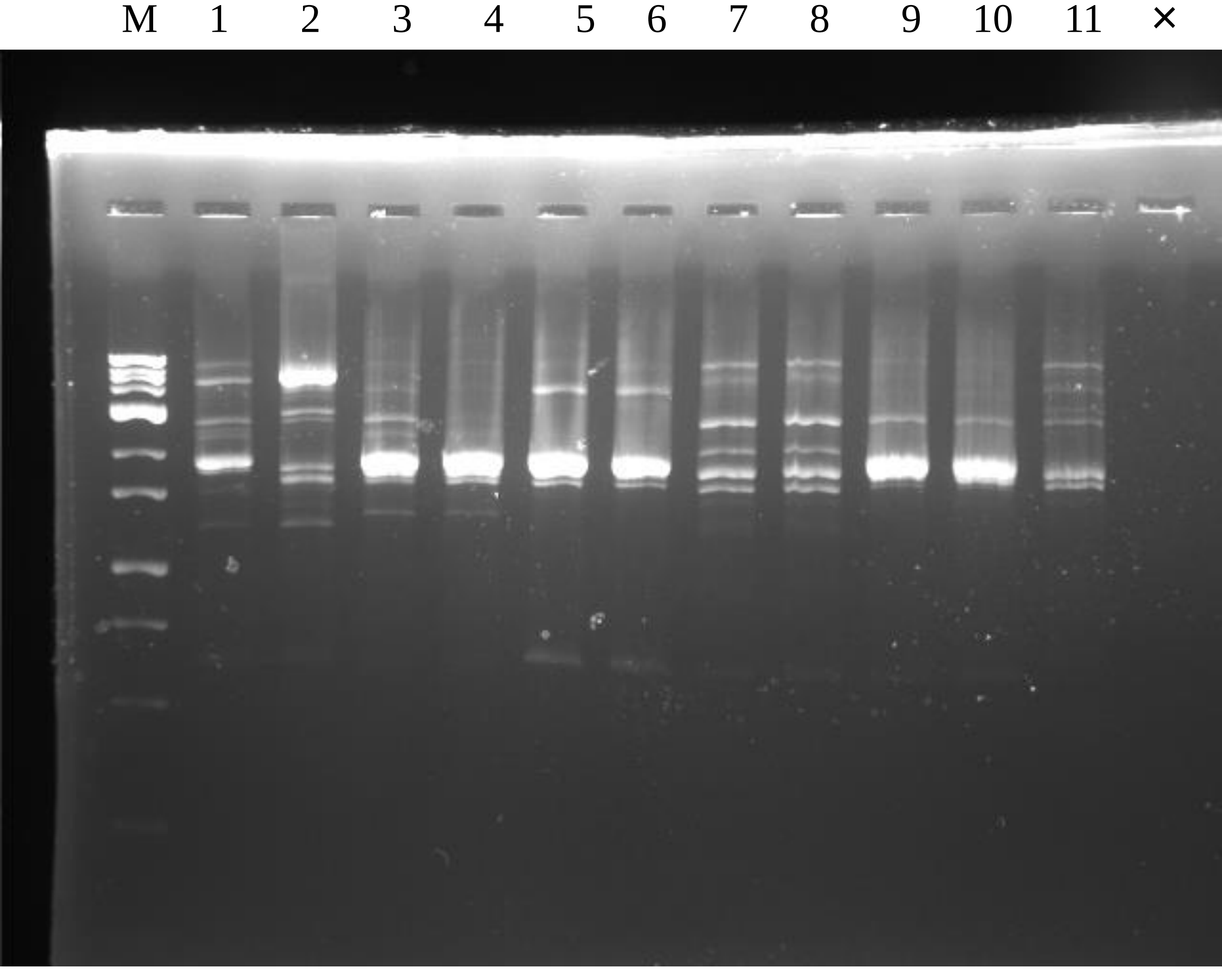

Figure 4A
Pc_1
DNA: 13
Primer: 02

× × × × × × × × × × × × × 13 ×

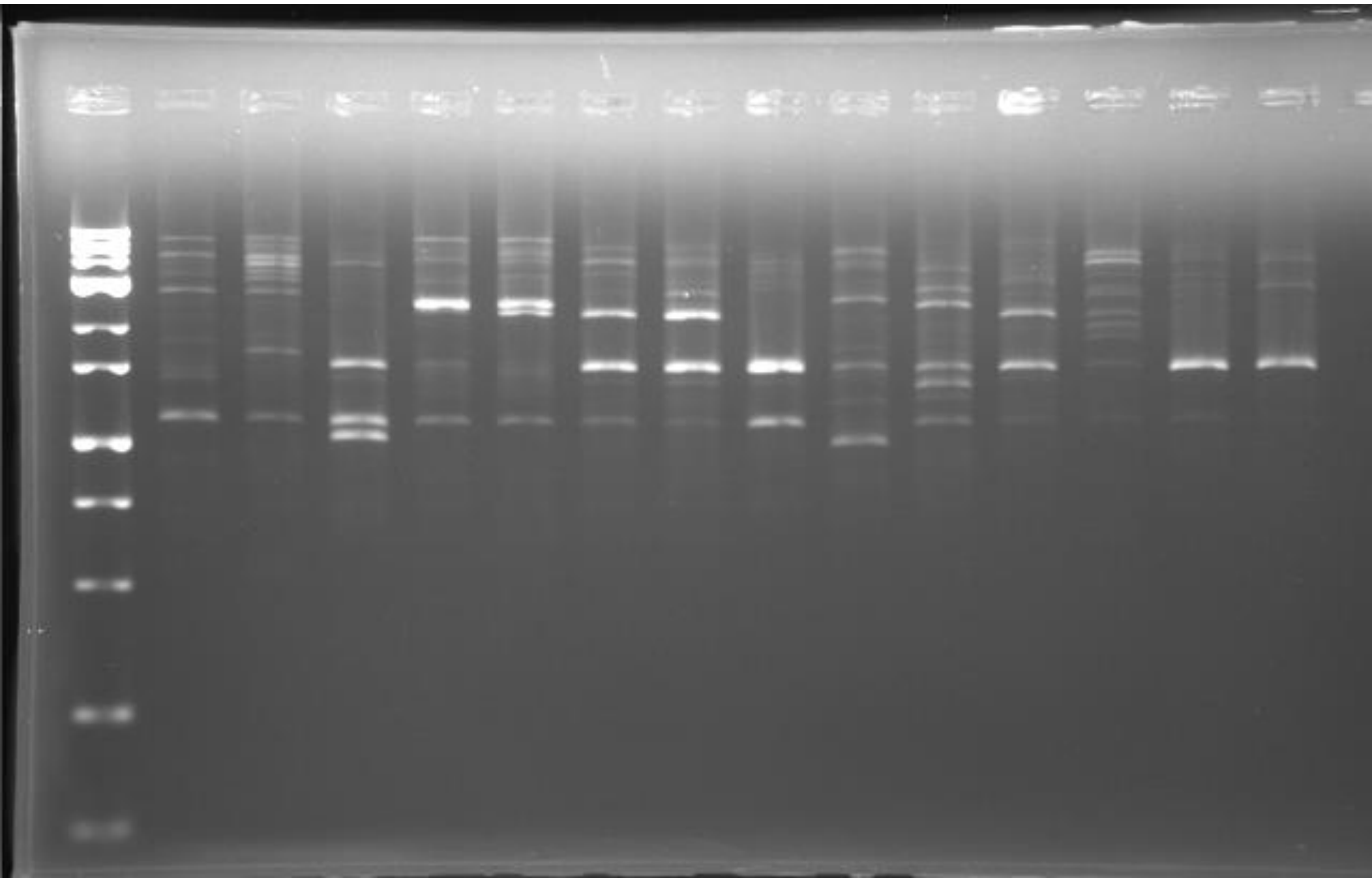

Figure 4A
Pc_2
DNA: 12
Primer: 05

× × × × × × × × × × × × 12 × ×

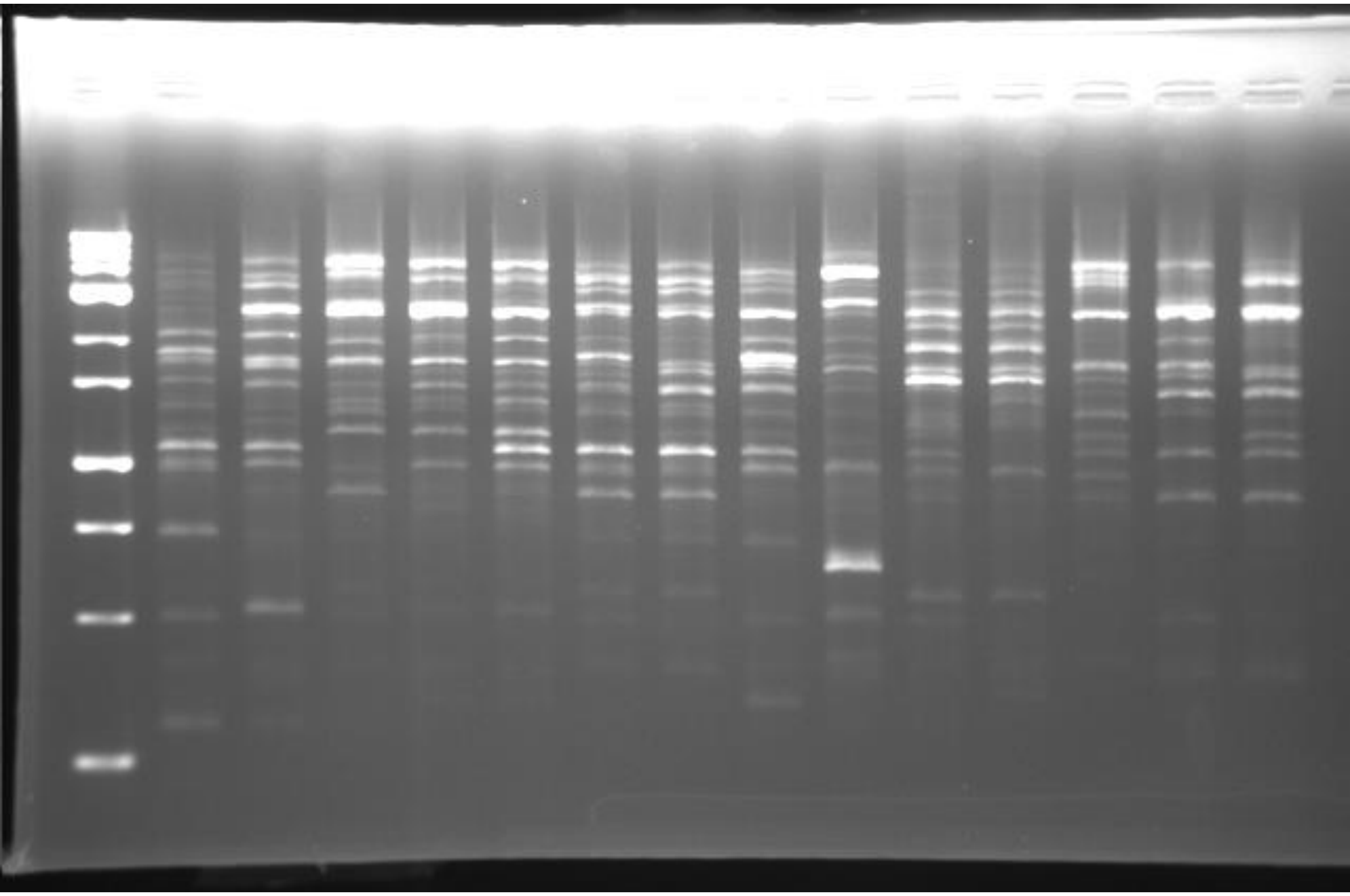

Figure 4A
Pc_3
DNA: 12
Primer: 02

× × × × × × × × × × × × 12 × ×

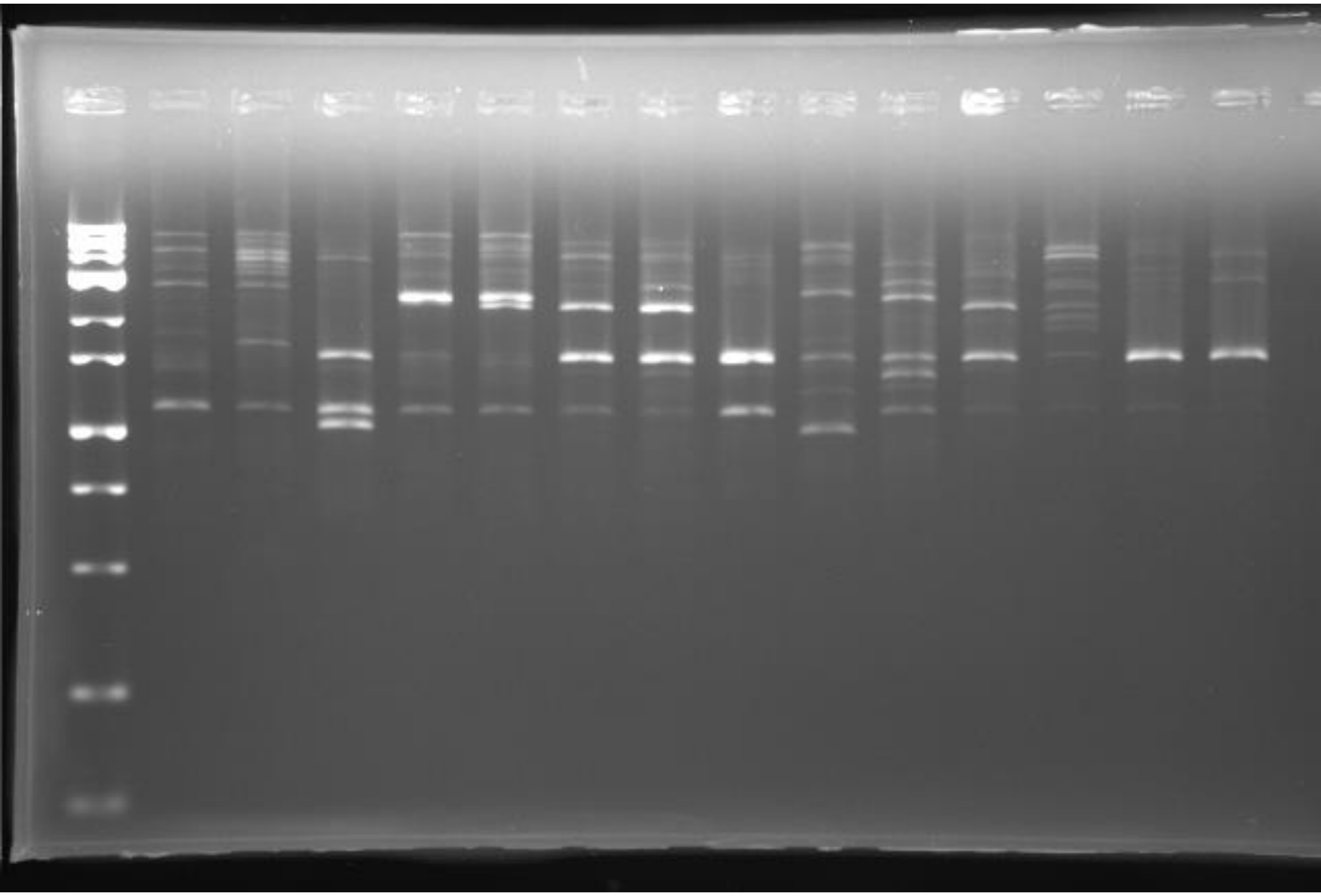

Figure 4A
Pc_4
DNA: 13
Primer: 04

× × × × × × × × × × 13 ×

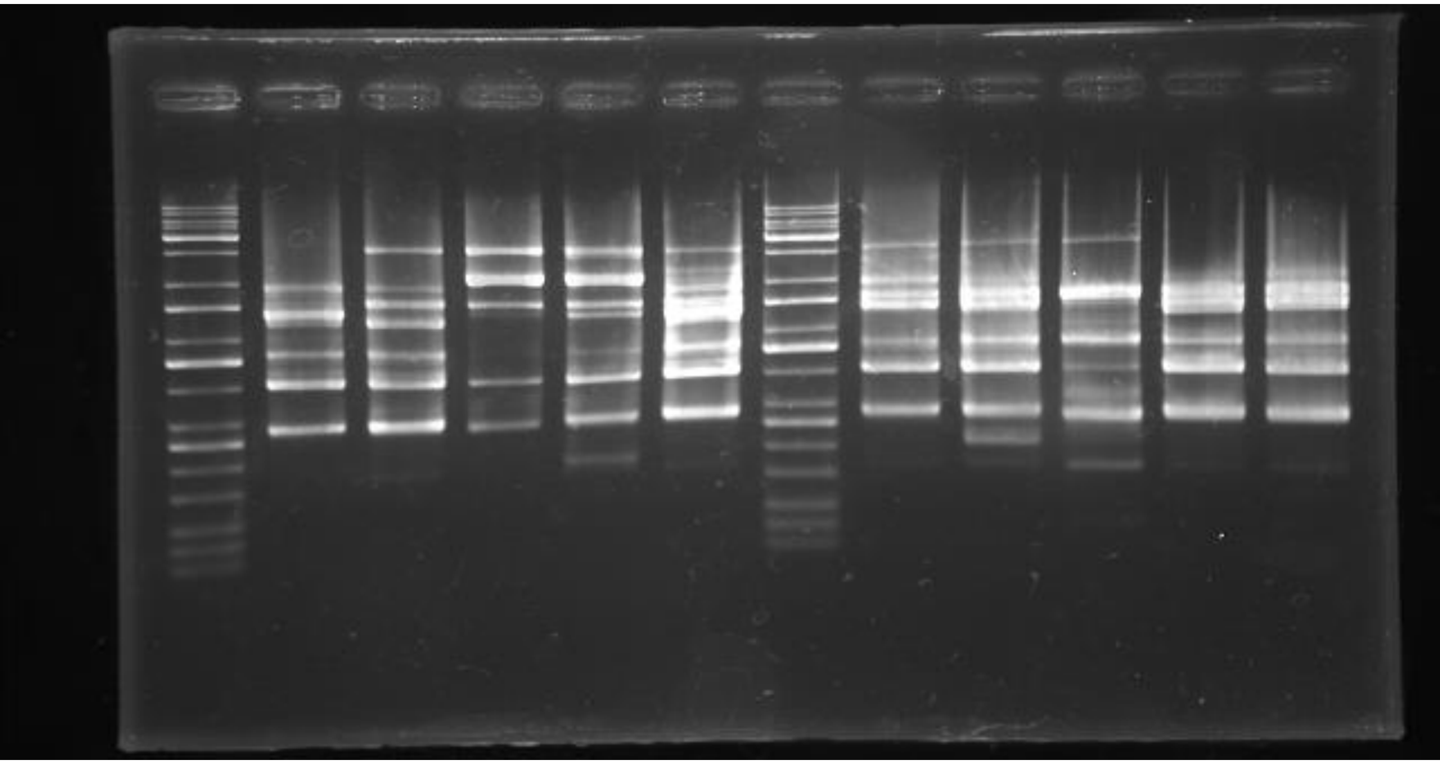

Figure 4A
Pc_5
DNA: 13
Primer: 09

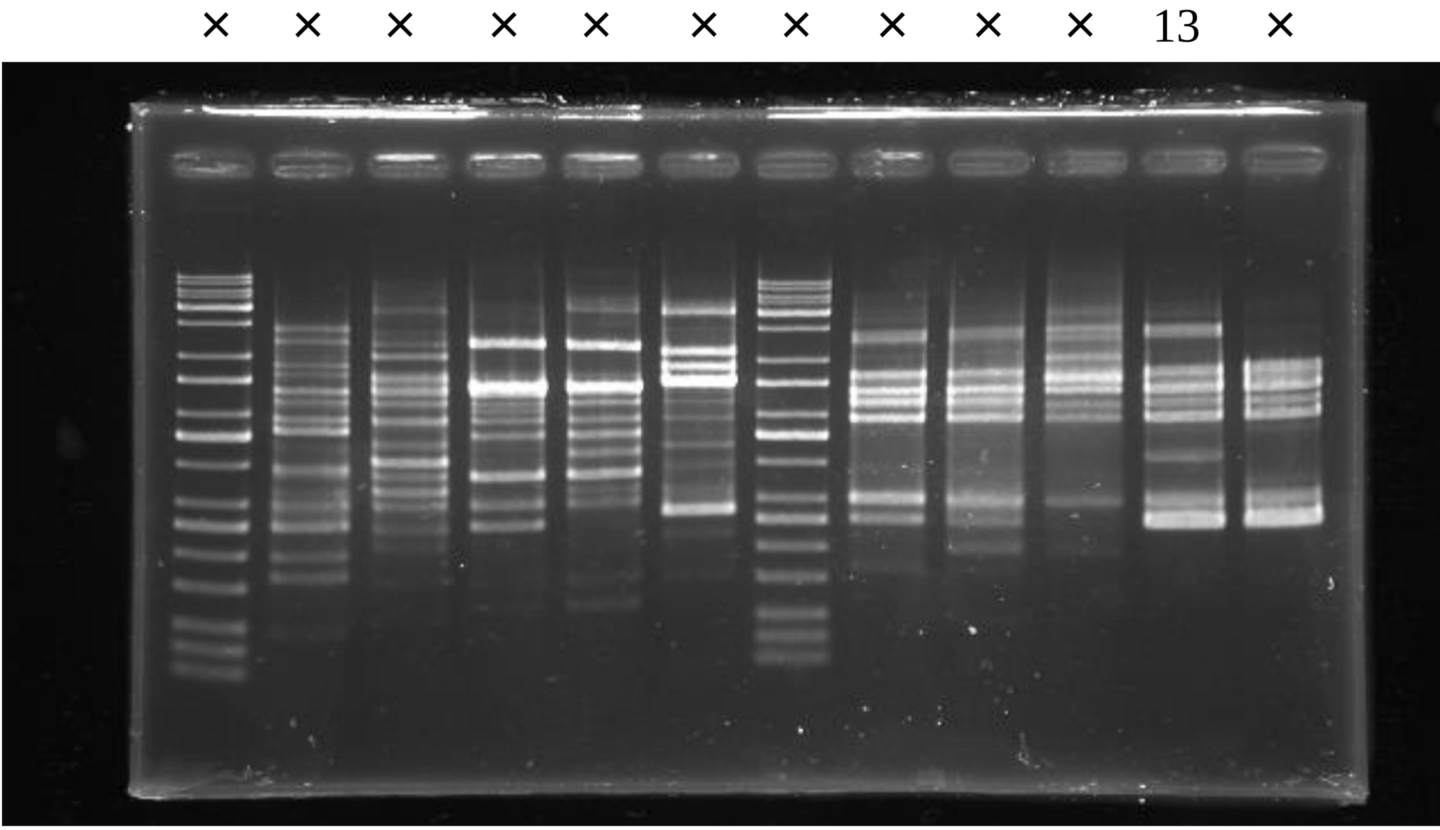

Figure 4B
Primer: Pc_1

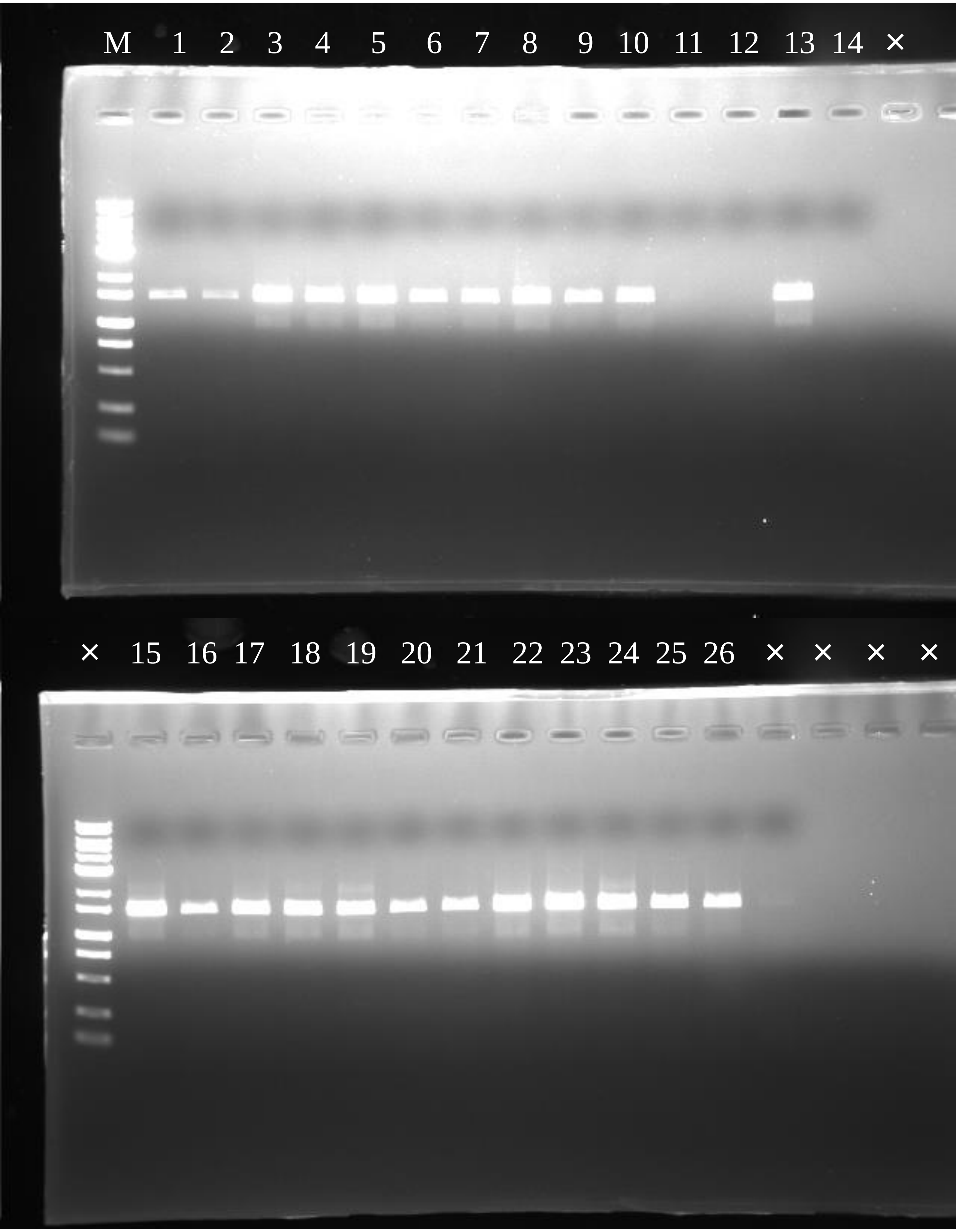

Figure 4B
Primer: Pc_2

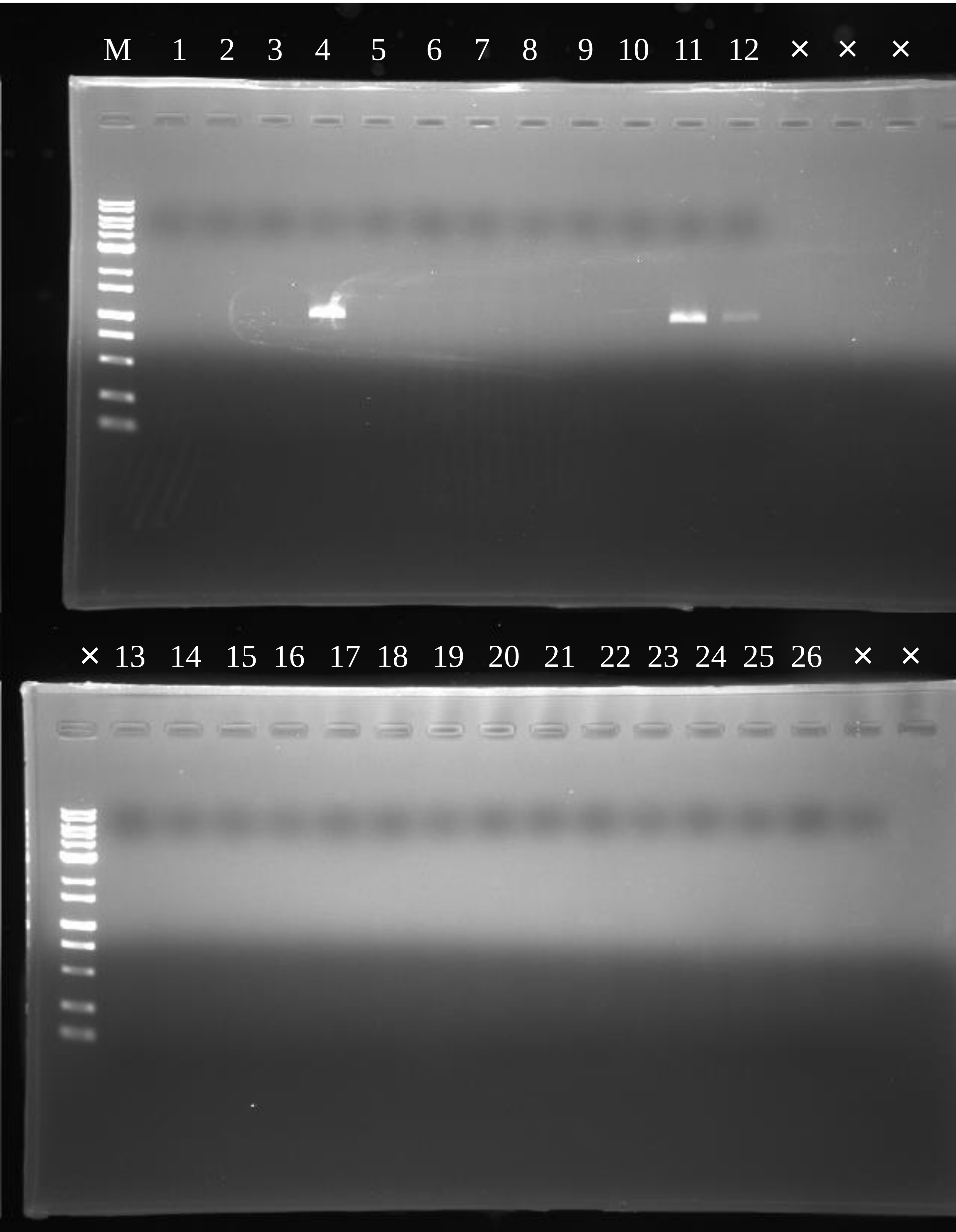

Figure 4B
Primer: Pc_3

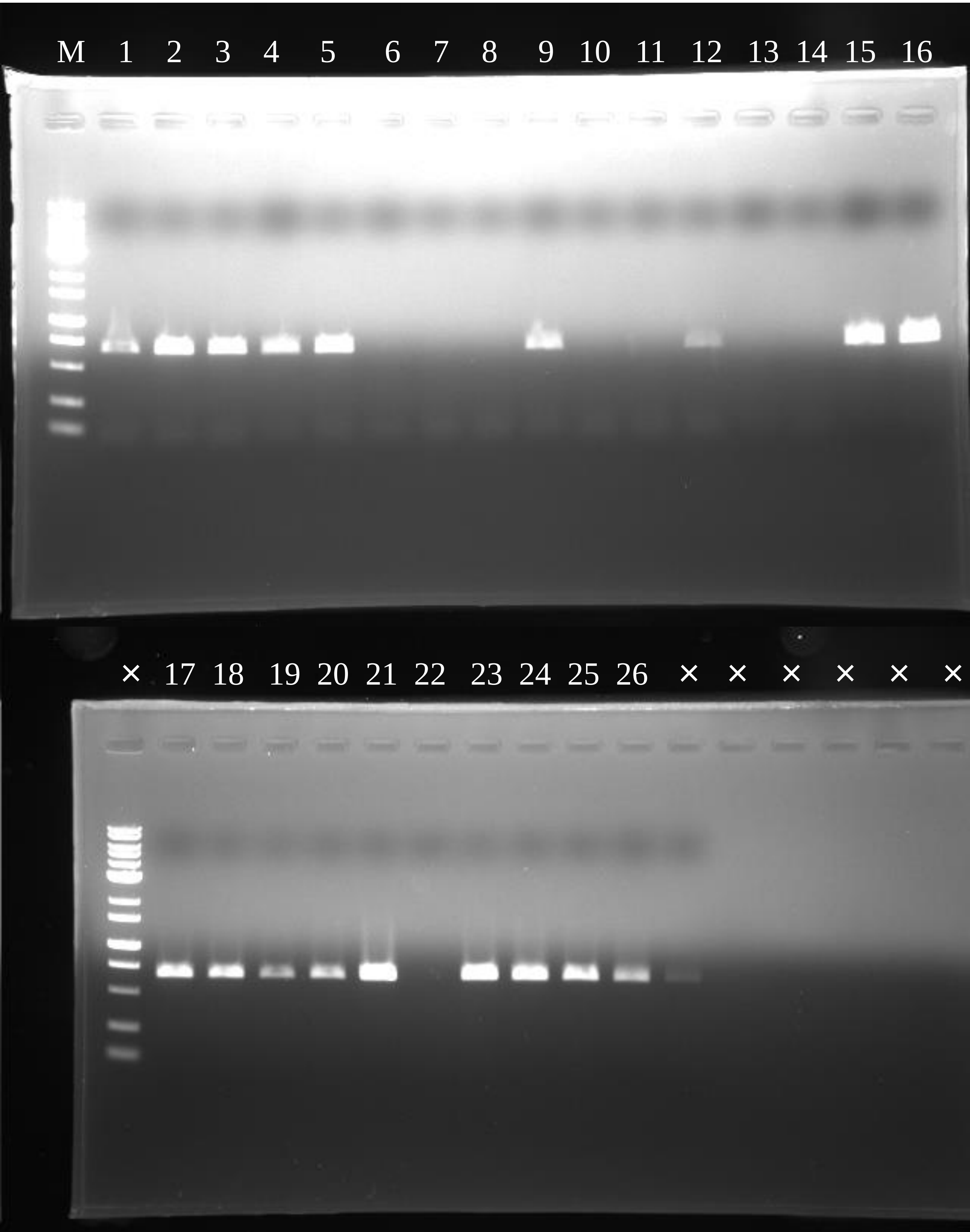

Figure 4B
Primer: Pc_4

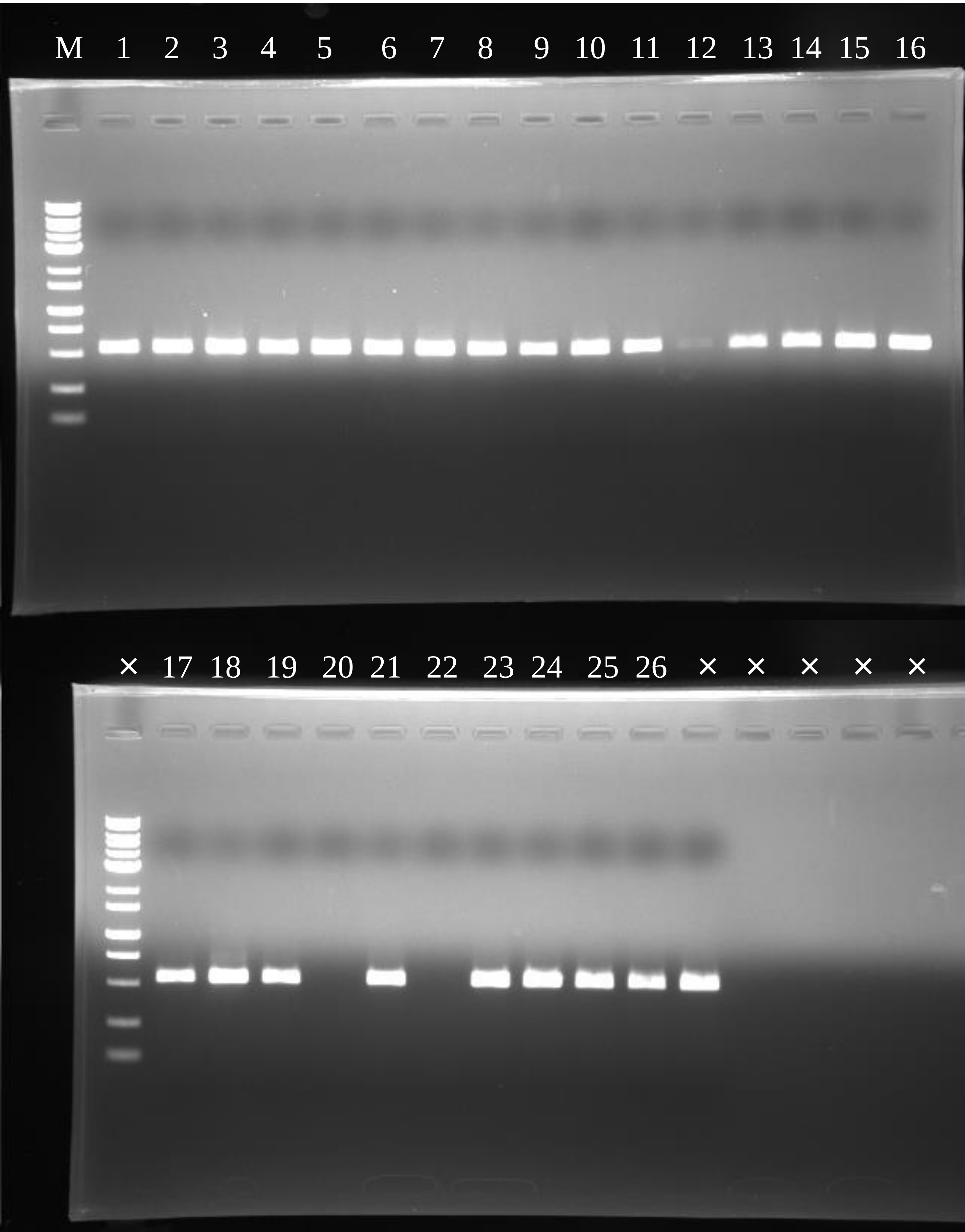

Figure 4B
Primer: Pc_5

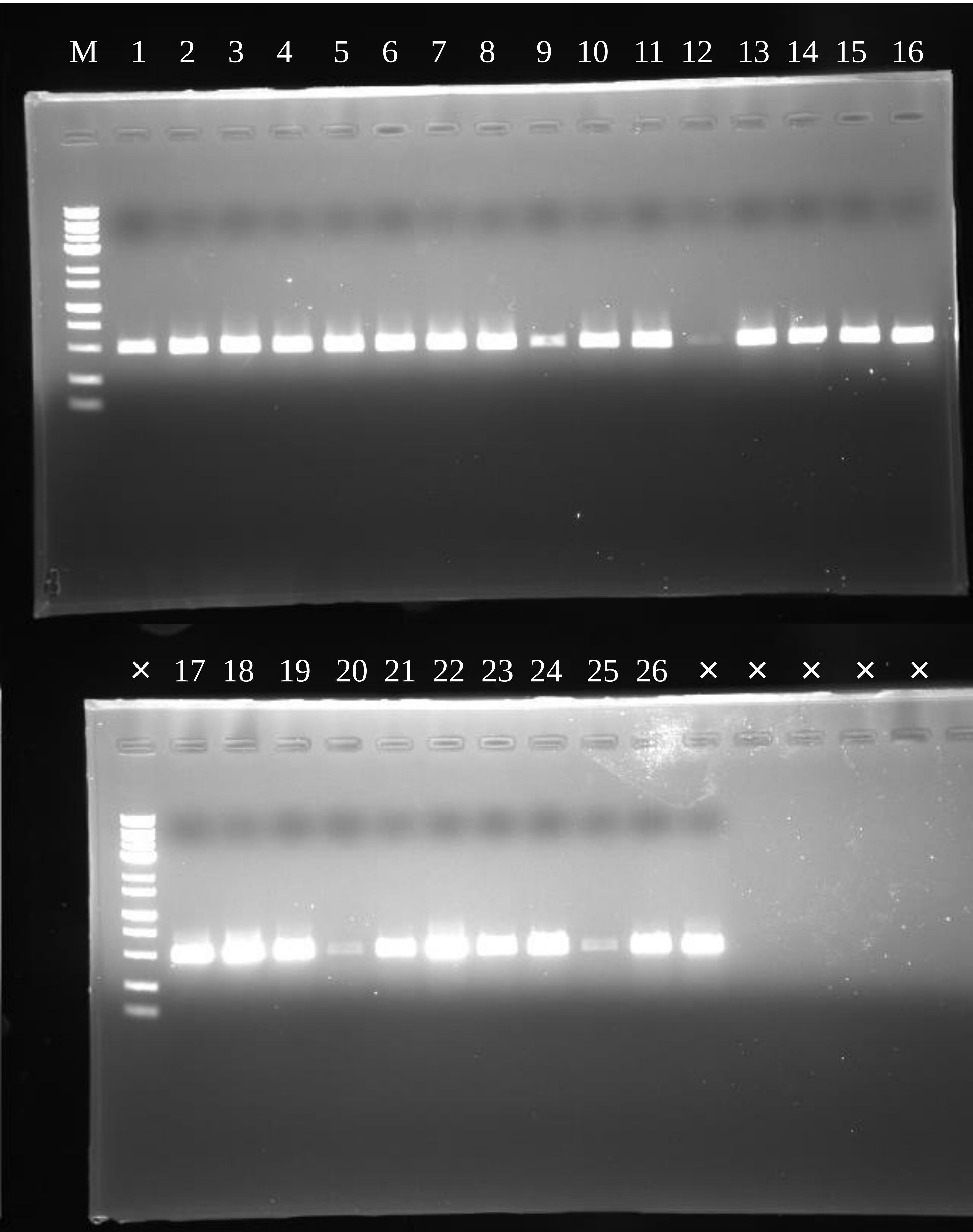

Figure 4B
Primer: 18S

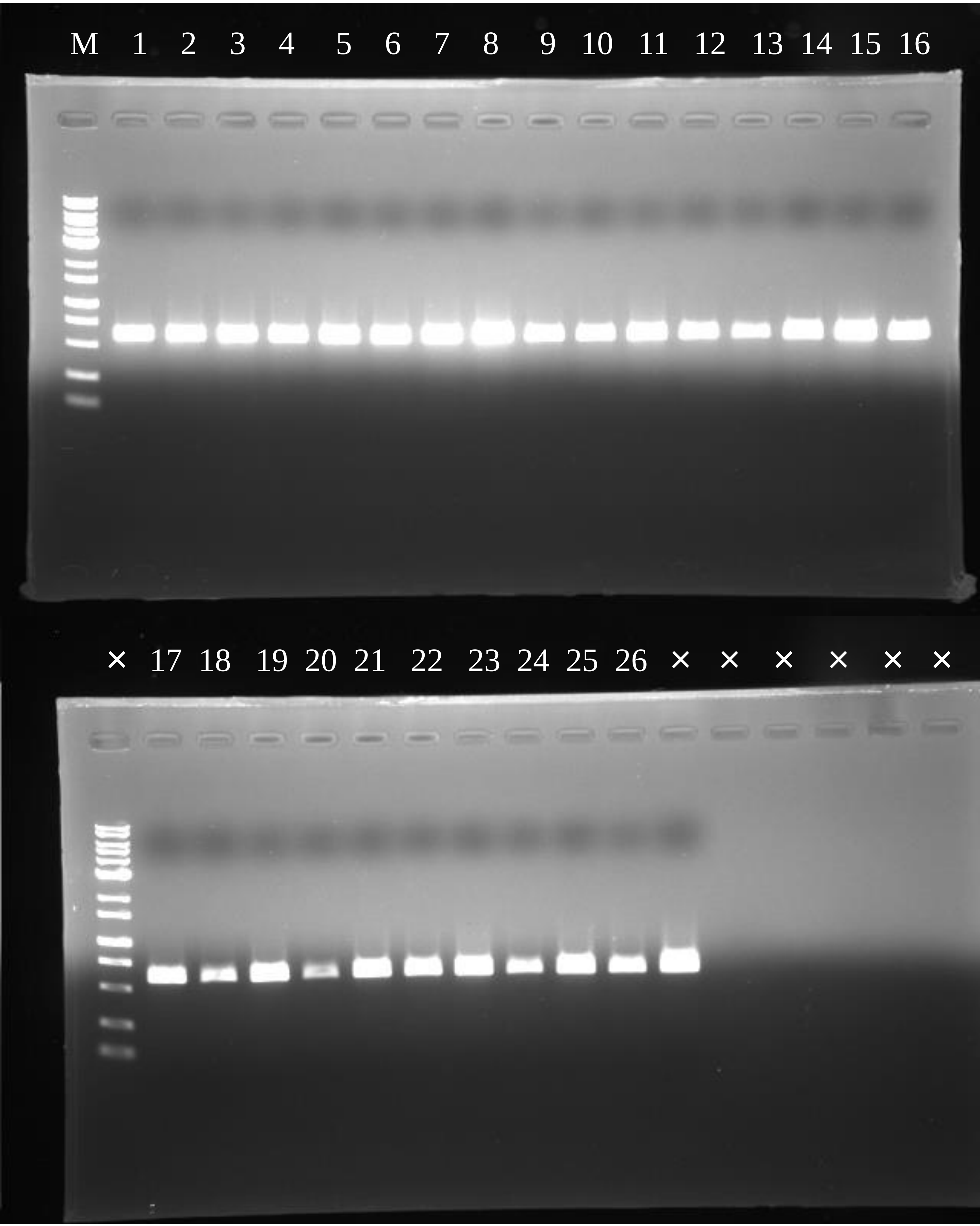

Figure 5

Primer: Pc_1, 2, 3, 4, 5 (multiplex)

M 4 5 11 12 13 14

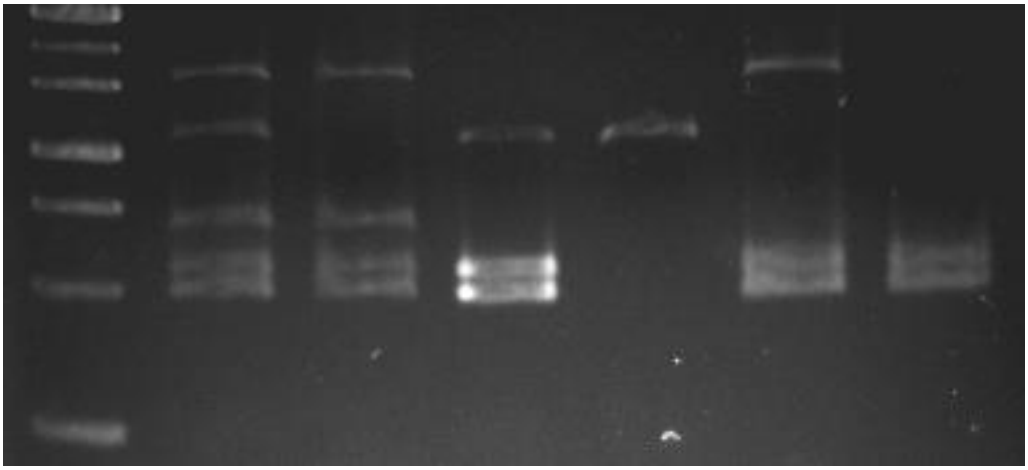

Supplement: S1 Raw images — (PDF) [file pone.0265139.s003.pdf]
